# Systemic Implications of Bullous Pemphigoid: Bridging Dermatology and Internal Medicine

**DOI:** 10.3390/diagnostics14202272

**Published:** 2024-10-12

**Authors:** Emi Mashima, Natsuko Saito-Sasaki, Yu Sawada

**Affiliations:** Department of Dermatology, University of Occupational and Environmental Health, Kitakyushu 807-8555, Japan; wahrheit25@yahoo.co.jp (E.M.); natsuko-saito@med.uoeh-u.ac.jp (N.S.-S.)

**Keywords:** bullous pemphigoid, BP180, Th2, Th17, systemic inflammatory response syndrome

## Abstract

**Background**: Bullous pemphigoid is an autoimmune bullous disease that frequently affects a large skin surface area, but it can also present in localized areas. It has been hypothesized that bullous pemphigoid affects the systemic functioning of different organs because inflammatory cells and cytokines circulate throughout numerous organs. **Results**: Recent clinical and experimental studies have revealed an association between bullous pemphigoid and systemic organ disorders. To avoid the emergence of systemic organ diseases, the significance of systemic treatment in cases of severe bullous pemphigoid should be emphasized. **Conclusions**: Here, we discuss the specific molecular processes underlying typical systemic organ inflammatory diseases associated with bullous pemphigoids.

## 1. Introduction

The interface between the external and internal environments is essential for the human body [1,2]. The skin serves as a biological defense system, not only protecting the body from harmful changes in the external environment [3,4] and infections [5,6] but also preventing excessive water loss [7,8], thereby maintaining the body’s hydration and homeostasis. The skin and mucous membranes physically divide the inside and outside of the body to prevent foreign substance invasion.

The role of type XVII collagen (BP180) is well understood in cutaneous hemidesmosomes, which promote basal keratinocyte adhesion and are found in high concentrations in skin epithelial cells [9] and the cervix [10], suggesting a possible influence of anti-BP180 antibodies on the development of a wide spectrum of human diseases.

Bullous pemphigoid (BP) is a representative blister disease that causes subepidermal blisters and is mediated by an autoantibody reaction against epidermal basement membrane antigens, particularly BP180, a hemidesmosome protein. Tense blisters and pruritus are clinically observed in skin and mucosal lesions. Pathogenic autoantibodies in bullous pemphigoid primarily target the epitopes located in the NC16a region of the transmembrane protein BP180 [11,12].

According to recent studies, systemic inflammatory diseases, including cardiovascular diseases, can develop from inflammatory skin conditions. Therefore, the presence of these risks and consequences should be considered in therapeutic settings. Moreover, representative inflammatory skin diseases such as psoriasis and atopic dermatitis enhance systemic inflammatory reactions and, in some cases, tissue or organ dysfunction [13,14]. Recent studies have determined the risk of inflammatory diseases and the pathophysiology of malfunction. This review highlights the specific risk factors and molecular processes caused by BP to emphasize the connection between skin inflammation in BP and systemic organ involvement.

## 2. Pathogenesis

**Figure 1:**
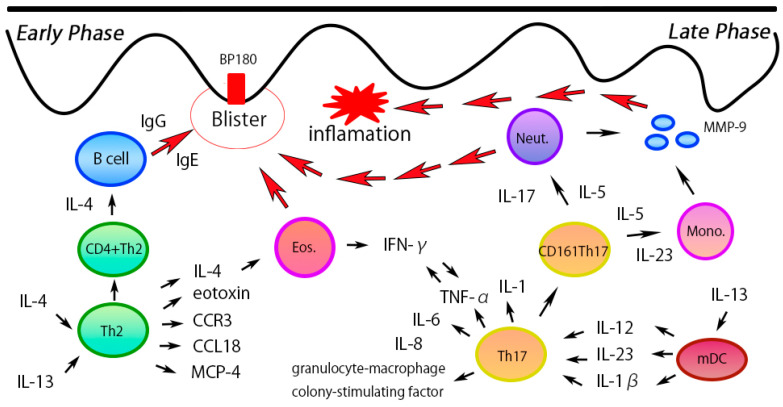
The pathogenesis of BP and its mediated immunological reactions. Autoantibodies that target BP180 and BP230 in bullous pemphigoid are associated with blister development in BP. In addition, Th2- and TH17-dominant immunological reactions also contribute to the development of the pathogenesis of BP.

The epidermis and dermis are intricately connected by hemidesmosomes and related proteins, and BP is triggered by immune-mediated damage to these anchoring structures (Figure 1). Autoantibodies targeting BP180 and BP230 play a key role in blister formation, but the precise mechanisms driving their production remain not fully understood.

Th2 cells predominantly contribute to humoral immunity by secreting cytokines such as IL-4, IL-5, IL-10, IL-13, and IL-15. In BP, there is significant infiltration of eosinophils and CD4+ T cells in the upper dermis, and Th2-related cytokines such as IL-4, IL-5, CCL18, and eotaxin have been detected at elevated levels specifically within BP skin lesions [15,16,17]. IL-4 and IL-5 are crucial for B cell activation and eosinophil recruitment [18], while IL-13 promotes IgE production [19,20], which correlates with disease severity. Additionally, IL-15 plays an essential role in eosinophilia and lesion severity through its interaction with IL-5 and IL-13 [21]. In BP, both eosinophils and IgE play critical roles in the immunopathogenesis of the disease [22,23]. IgE autoantibodies are believed to contribute to the activation of mast cells and basophils, leading to the release of pro-inflammatory mediators that exacerbate the condition [22]. Furthermore, eosinophils are highly abundant in BP skin lesions and are thought to contribute to tissue damage and blister formation through the release of cytotoxic granules and cytokines [24,25]. In addition to their direct role in blister formation, eosinophils also contribute to the activation of the Th2-mediated inflammatory pathway by releasing cytokines such as IL-4, IL-5, IL-13, CCL5, and TGF-β. These cytokines not only promote further recruitment and activation of eosinophils but also enhance Th2 cell activation, thus amplifying the inflammatory cascade in BP. Several studies have demonstrated that elevated serum IgE levels are often associated with disease severity in BP, further supporting the involvement of IgE in the systemic inflammatory cascade [26]. Similarly, eosinophils, through their activation and degranulation, have been shown to play a key role in driving the inflammatory response in BP, contributing to tissue injury and promoting blister formation [27].

Th17 cells and their associated cytokines, particularly IL-17 and IL-23, are central to the type 3 inflammatory response observed in BP. Th17 cells infiltrate BP skin lesions, and IL-17 has been shown to stimulate neutrophil recruitment and matrix metalloproteinase-9 (MMP-9) production [28], contributing to the separation of the dermo-epidermal junction (DEJ) and blister formation [29]. Although IL-17 levels in BP serum are not directly correlated with disease severity, elevated IL-17 and IL-23 levels are associated with relapse risk and may influence resistance to glucocorticoid therapy through the upregulation of glucocorticoid receptor β (GR-β) [30,31].

The pathogenesis of BP involves a complex balance between Th2 and Th17 responses. Th2 cytokines, such as IL-4 and IL-13, drive eosinophil activation and IgE production [32,33], both of which are critical in tissue damage and blister formation [34,35]. Th17 cytokines, such as IL-17 and IL-23, play a pivotal role in neutrophil recruitment and extracellular matrix degradation [36,37]. Together, these immune responses create a positive feedback loop, maintaining the hyperinflammatory state observed in BP.

## 3. BP and Systemic Organ Diseases

**Figure 2:**
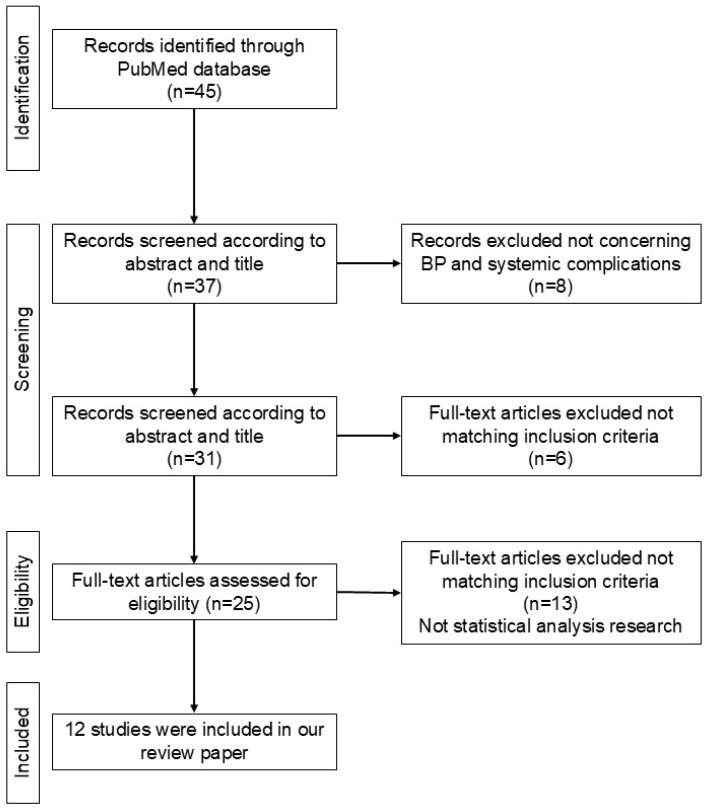
PRISMA flow diagram for literature review.

BP is associated with cancer and systemic organ diseases. This section provides an overview of systemic organ diseases associated with cerebrovascular and cardiovascular diseases, renal dysfunction, Alzheimer’s disease, ulcerative colitis, thromboembolism, and bone fractures. To comprehensively assess the association between BP and various systemic complications, we performed a search on PubMed using the terms ‘bullous pemphigoid’ combined with various systemic complications such as cardiovascular diseases and other relevant conditions. Studies were included if they reported on the association between BP and these systemic complications, provided hazard ratios or related statistical measures, and were published in peer-reviewed journals (Figure 2).

This flowchart illustrates the process of selecting studies for inclusion in the review paper. A total of 45 records were initially identified through the PubMed database. After screening by title and abstract, 37 records were eligible for further review, with 8 being excluded for not addressing BP and systemic complications. After further screening, 31 records remained, and 6 more were excluded for not matching the inclusion criteria. Subsequently, 25 full-text articles were assessed for eligibility, leading to the exclusion of 13 that did not meet the statistical analysis requirements. Finally, 12 studies were included in the review.

## 4. Bullous Pemphigoid and Cardiovascular Disease

Myocardial or cerebrovascular events are lethal emergency conditions caused by coronary or cerebral artery blockage, atherosclerotic plaque rupture, or arterial spasms [38]. Recent studies in various countries have shown the important risk factor for cardiovascular disease. A study conducted in Taiwan among 390 individuals found that over a 3-year observation period, 22.8% of patients with BP experienced a stroke, compared to 11.4% of individuals without BP [39]. The hazard ratio (HR) for stroke was 2.37 (95% confidence interval [CI]: 1.78–3.15) times higher in people with BP compared with those without BP [39]. Another retrospective cohort analysis that included 359 newly diagnosed patients with BP in a Singapore population showed a high frequency of cardiovascular (14.6%) and stroke (11.6%) events; these events were the leading causes of death in this population [40]. Patients with BP had greater cardiovascular disease mortality at 1 year than matched controls (7.9% and 1.3%, respectively), according to a cohort analysis of 252 patients with BP in the Taiwanese population [41]. At 1 year, patients with BP had a five-times-higher increased risk of death associated with cardiovascular disease (HR: 5.3, 95% CI: 2.4–11.7) [41]. Furthermore, all-cause mortality associated with BP was greater in individuals who had not previously used corticosteroids (HR: 5.7, 95% CI: 4.2–7.6) [41]. A study conducted in Europe reported a higher risk of cardiovascular disease in patients with BP. A study of 198 cases of BP in a Finnish population also revealed that the most prevalent comorbidity (76.3%) was cardiovascular events [42].

The immunological phenotypes of BP pathogenesis may contribute to the development of cardiovascular disease in patients with BP. The endothelial inflammatory response induces the formation of atherosclerotic plaques [43]. Patients with coronary atherosclerosis were demonstrated to have a higher number of circulating IL-17-producing cells in the peripheral blood and cultured atherosclerotic coronary arteries [44]. Endothelial dysfunction is exacerbated by epidermal IL-17A overexpression in a mouse experiment [45]. Additionally, an enhanced Th2 immune response is associated with vascular inflammation. Dupilumab therapy adversely controls genes associated with atherosclerosis in Th2-dominant illnesses, indicating the potential significance of systemic treatment for BP for the negative regulation of pro-inflammatory cytokine production in vascular inflammation in a human experiment [46]. However, it is essential to recognize that other treatments, such as corticosteroids, are commonly used for BP management and are effective in controlling disease activity [47]. Although corticosteroids do not have evidence supporting their impact on cardiovascular events, they remain a crucial component of BP treatment strategies. A comprehensive approach to therapy should consider the full range of available options and their respective roles.

## 5. Renal Dysfunction

Chronic kidney disease (CKD) is characterized by a persistent modification in kidney structure and function [48] and recent studies have identified BP as a possible risk factor for CKD.

A cohort study of 91 patients with BP found that these patients had elevated CKD (odds ratio [OR]: 2.3, 95% CI: 1.2–4.4) and end-stage renal disease (ESRD) (OR: 3.8, 95% CI: 1.5–9.9) risks [49]. A prior hemodialysis administration in patients with ESRD delayed the BP onset [50,51].

BP-related inflammatory immune reactions affect the immune response in the kidney, which is a factor in the development of renal failure. The frequencies of Th17 and Th2 cells were shown to be higher in patients with ESRD [52]. IL-17A-producing cells are associated with the production of inflammatory cytokines and determine the degree of renal injury. The inhibition of IL-17A improved renal dysfunction [53]. IL-17A-deficient mice have the potential to prevent renal damage, suggesting that a blockage of IL-17A leads to the downregulation of renal dysfunction in BP [54].

IL-4 induces renal fibrosis [55]. The frequency of IL-4-producing cells in peripheral blood was higher in a mouse model of CKD, and IL-4 deficiency resulted in Myd88-mediated renal fibrosis in a mouse experiment [56]. Furthermore, IL-33 is involved in the development of CKD. Serum IL-33 levels are also elevated in patients with CKD [57]. Therefore, the Th2 immune response might contribute to the pathophysiology of CKD. These findings suggest a strong link between BP and CKD, with immune dysregulation, particularly involving Th17 and Th2 pathways, playing a key role in renal damage. The involvement of cytokines such as IL-17A and IL-4 in renal inflammation and fibrosis highlights the importance of targeting these pathways in managing both BP and its renal complications. Further research is necessary to explore the therapeutic potential of IL-17A and IL-4 inhibition in preventing CKD progression in BP patients.

## 6. Alzheimer’s Disease and BP

Alzheimer’s disease is a common cause of acquired memory loss in middle and late life. Amyloid (A)-containing extracellular plaques and tau-containing intracellular neurofibrillary tangles are the two main contributors to the development of neurodegenerative symptoms in Alzheimer’s disease [58].

The prior existence of a neurological disorder, such as Alzheimer’s disease, is a risk factor for the future development of BP. A study of 87 patients with high blood pressure in the United States showed a higher BP risk in patients with a history of any neurologic disorder (OR: 6.9; 95% CI: 3.0–15.6) or dementia (OR: 6.8; 95% CI: 2.1–21.9) [59].

In a study, patients with Alzheimer’s (18%) showed a higher frequency of BP180 autoantibodies compared with control participants (3%) [60]. On indirect immunofluorescence examination, none of the sera of patients with Alzheimer’s were positive for the full-length human anti-BP180 autoantibody, which responds to the basement membrane in the skin [60]. Additionally, higher BP180-NC16A autoantibody levels in the cerebrospinal fluid are associated with cognitive impairment [60]. A Danish population of 3281 patients with BP also had an elevated risk of Alzheimer’s disease (OR: 27.0) [61]. A retrospective cross-sectional analysis of 1743 individuals with BP in the German population found that Alzheimer’s disease was related to the prior existence of BP (OR: 2.1, 95% CI: 1.7–2.6) [62].

A high frequency of serum response with human cutaneous BP180 protein was observed in 39.1% of patients with Alzheimer’s disease (39.1%) [63] and the serum from patients with Alzheimer’s disease also showed a high positive frequency response with BP180 protein in the extract from the human brain (47.8%) [63]. Therefore, the anti-BP180 antibody may directly influence the development of Alzheimer’s disease. In addition, patients with Alzheimer’s disease show a high frequency of Th17 cells, which have been postulated as a possible cause of Alzheimer’s disease [64]. Alzheimer’s, induced by injecting amyloid 1-42 (A1-42) into the hippocampus, enhances the infiltration of Th17 cells into the brain [65] and increased IL-17 and IL-22 levels in the hippocampus in a mouse experiment [65]. Th17 cells are the main source of FAS ligand expression, which induced neuronal death in a mouse experiment [65]. Additionally, anti-IL-17 antibody treatment reduces inflammatory cytokine production and A1-42-induced neurotoxicity, having resulted in beneficial effects on memory function in a mouse experiment [66]. Adalimumab also impairs the development of Alzheimer’s disease by suppressing NF-κB, a crucial component of neuroinflammatory transcription factors, suggesting that BP-mediated inflammatory reactions might be a potential therapeutic candidate for the impairment of Alzheimer’s disease development [67]. These findings suggest a significant interplay between BP and Alzheimer’s disease, with BP180 autoantibodies and Th17-mediated inflammation potentially contributing to both conditions. The shared immunological mechanisms, including the role of IL-17 and NF-κB, highlight a possible link between skin and neurodegenerative disorders.

## 7. Ulcerative Colitis and BP

Previous case studies suggested that BP may be associated with the development of ulcerative colitis [68,69]. Population-based research of 5263 BP cases in the Taiwanese population found that those with BP had a greater prevalence of ulcerative colitis (0.38%) compared with healthy subjects (0.16%) [70] and an increased risk of ulcerative colitis (OR: 3.6, 95% CI: 1.9–6.8) [70].

IL-17 may play a positive role in the development of intestinal inflammation [71]. Additionally, inflammation in colitis is exacerbated by IL-4. Dextran sulfate sodium-induced colitis in mice was hampered by IL-4 deficiency in a mouse experiment [72]. These findings suggest that BP might be involved in the development of ulcerative colitis in some parts. These findings indicate a potential link between BP and ulcerative colitis, with immune dysregulation, particularly involving IL-17 and IL-4, likely contributing to this association. The role of these cytokines in promoting intestinal inflammation suggests that patients with BP may be at increased risk for developing ulcerative colitis.

## 8. Thromboembolism and BP

BP is associated with a high risk of deep venous thromboembolism (DVT). A total of 12,692 patients with BP in Taiwan had a substantially higher frequency of venous thromboembolism (0.2%) compared with the non-BP cohort (0.1%) and increased risk of venous thromboembolism (HR: 2.02, 95% CI: 1.0–4.1) [73]. Another cohort analysis of 432 patients with BP revealed a higher incidence rate (56.7 [95% CI: 33.0–80.4]) of venous thromboembolism (per 1000 patient years) during the acute phase compared to the remission phase (6.3 [95% CI: 2.8–11.3]) [74], suggesting the risk of thromboembolism depending on the phase of BP.

Although the detailed mechanism of DVT occurrence in patients with BP remains unknown, IL-17A is known to be involved in DVT formation. It has been reported previously that IL-17 levels increase in patients with DVT [75] and in a disease mouse model [76]. Recombinant IL-17A administration exacerbates the development of DVT; this effect can be nullified by blocking IL-17, a step mediated by enhanced platelet activation and aggregation, neutrophil infiltration, and EC activation in a mouse experiment [76]. Hence, anti-IL-17A mAb could be used for the treatment of DVT [76].

The association between BP and an elevated risk of DVT underscores the systemic nature of BP, which extends beyond the skin to involve other organs and physiological systems. Although the exact mechanisms driving this increased risk of DVT remain unclear, current evidence suggests a multifactorial process involving both systemic inflammation and immune dysregulation. IL-17A, which has been implicated in various inflammatory diseases, appears to play a critical role in DVT pathogenesis, as elevated IL-17A levels have been observed in both clinical settings and experimental models of DVT. The pro-inflammatory effects of IL-17A, including enhanced platelet activation, neutrophil recruitment, and endothelial cell activation, create a thrombogenic environment that may explain the higher incidence of thromboembolism in BP patients.

## 9. Bone Fracture

Osteoporosis is characterized by decreased bone mass, bone tissue degeneration, and increased bone fragility [77]. Bone remodeling is the process of replacing old bone to regenerate the skeleton. Following the recruitment of osteoclasts, mineralized bone is resorbed, and osteoblasts are recruited to the site to produce mineralization of new bone. Osteoporosis-related bone fractures are becoming more prevalent in older populations [77] and an increased risk of osteoporosis or bone fractures has been observed in patients with BP. A cross-sectional study including 8864 patients with BP showed that BP was related to an increased risk of osteoporosis (OR: 1.6, 95% CI: 1.4–1.7) and pathological fractures (OR: 1.5, 95% CI: 1.2–1.9) [78]. In addition, vitamin D deficiency increases the risk of bone fractures. Fifteen patients with BP had lower serum 25-hydroxyvitamin D levels and a higher frequency of lower vitamin D levels [79]. The increased risk of osteoporosis and fractures in patients with BP appears to be accelerated by glucocorticoid use, as studies have accounted for this factor statistically. The chronic inflammatory state associated with BP, along with vitamin D deficiency, likely plays a significant role in reducing bone density and increasing fragility.

## 10. Whole Interaction of BP with Systemic Organ Inflammation

**Figure 3:**
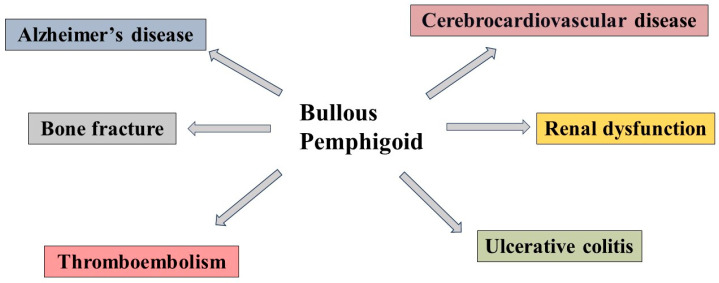
The interaction of immunological reactions derived from BP to other systemic organs. BP has been linked to the occurrence of systemic organ inflammation. This figure provides an overview of some systemic inflammatory diseases that are connected to cerebrocardiovascular diseases, renal dysfunction, Alzheimer’s disease, ulcerative colitis, thromboembolism, and bone fracture.

Figure 3 shows the relationship between BP and other organ diseases. Because only a few studies have been undertaken to reveal the molecular-based processes of BP-mediated inflammation in the development of various organ dysfunctions, an undiscovered etiology for the development of BP-mediated systemic inflammatory diseases is expected to be clarified in the future. BP predominantly affects elderly patients, many of whom often have comorbid conditions, including diabetes mellitus [80,81]. The relationship between BP and diabetes remains complex and not fully elucidated. However, it is well-documented that systemic corticosteroids, a mainstay of BP treatment, can exacerbate hyperglycemia, particularly in individuals with pre-existing diabetes or those at risk of developing glucose metabolism disorders.

## 11. Conclusions

BP influences the development of inflammatory diseases and associates with various systemic inflammatory disorders. The inflammation of secondary systemic sites following BP might be suppressed by controlling the disease condition of BP and preventing the development of systemic organ diseases; therefore, the accurate evaluation of disease severity and the regulation of the disease control of BP is an important issue for clinicians to select the best therapeutic approach.

Systemic therapies for BP encompass a range of treatment options, including glucocorticoids, immunosuppressives (such as azathioprine, methotrexate, and mycophenolate mofetil), antibiotics (such as dapsone and tetracyclines), and biologic therapies (such as dupilumab, omalizumab, and rituximab) [82,83]. Glucocorticoids are the cornerstone of BP treatment, effectively controlling inflammation and reducing disease activity. Immunosuppressive agents, including azathioprine, methotrexate, and mycophenolate mofetil, are often used as adjuncts to glucocorticoids, particularly in cases of severe or refractory disease [84]. Antibiotics, such as dapsone and tetracyclines, offer an alternative or adjunctive therapy, especially in patients with contraindications to systemic steroids or immunosuppressives [85,86,87]. In recent years, biologic therapies have gained attention for their potential benefits in BP management. Dupilumab, an IL-4/13 inhibitor, is currently undergoing clinical trials for BP, which may offer new insights into its efficacy [88,89]. Other biologics, such as omalizumab (an anti-IgE antibody) and rituximab (a CD20-targeted monoclonal antibody), have been explored with varying degrees of success [90,91,92,93,94]. It is important to note that many of the assumptions about the potential benefits of systemic therapies for BP, particularly in preventing internal diseases, are derived from data on other inflammatory dermatoses, such as psoriasis [13,95]. These extrapolations should be interpreted with caution, as the specific effects of these therapies on BP and associated systemic complications require further investigation. Additionally, while IL-4/13 inhibition is being explored in clinical trials for BP, IL-17A blockade is not currently in clinical development for autoimmune blistering diseases, including BP. Notably, there are reports of paradoxical BP reactions associated with IL-17A inhibitors used for other conditions, such as psoriasis [96]. This highlights the need for careful consideration of potential adverse effects when applying therapies developed for other inflammatory conditions. Overall, while systemic therapies offer potential benefits for BP, their role in preventing internal diseases and managing BP remains an area of active research. Future studies should continue to explore these treatments’ effectiveness and safety profiles to better inform clinical practice.

Currently, systemic treatments for BP, such as corticosteroids and immunosuppressants, are effective in controlling the disease but often exacerbate pre-existing comorbidities, such as diabetes and cardiovascular diseases. For example, corticosteroids are known to induce hyperglycemia, which complicates blood glucose control in patients with diabetes [81]. Newer biologic agents like rituximab and dupilumab offer promise, as they have been shown to have fewer systemic side effects, potentially improving outcomes in patients with multiple comorbidities [89]. These biologics may also reduce the need for long-term corticosteroid use, thereby decreasing the risk of complications such as osteoporosis, infections, and cardiovascular events. Looking to the future, ongoing research into novel biologics targeting specific pathways, such as IL-17, IL-23, and complement inhibition, holds potential for safer and more targeted therapies. These therapies may provide better control over BP while minimizing the impact on blood glucose levels and other comorbid conditions. Additionally, emerging treatments like complement inhibitors are being investigated for their ability to control the immune response in BP, which could further reduce complications associated with systemic inflammation.

Drugs like gliptins (DPP-4 inhibitors), commonly prescribed for type 2 diabetes, have also been linked to BP. Several studies have suggested that gliptins can exacerbate or trigger BP, possibly through their effects on immune regulation. As many BP patients are elderly and often present with comorbidities such as diabetes, it is crucial to evaluate the impact of these drugs on disease severity and systemic involvement. Moreover, the use of PD-1 inhibitors, a class of immunotherapy drugs used in cancer treatment, has been associated with the development of BP as an adverse effect. These inhibitors, by unleashing the immune system to target cancer cells, may inadvertently contribute to autoimmune diseases like BP.

Determining the best treatment to control the emergence of systemic organ diseases is an issue. There are already many biological possibilities, and more clinical studies are needed to determine how these systemic organs manifest. As various skin inflammatory diseases are a potent initiator of systemic inflammatory illnesses, there may be unidentified organ impacts; hence, more research is necessary to understand these unidentified systemic influences.
